# Modeling the Piezoelectric Cantilever Resonator with Different Width Layers

**DOI:** 10.3390/s21010087

**Published:** 2020-12-25

**Authors:** Zhenxi Liu, Jiamin Chen, Xudong Zou

**Affiliations:** 1 School of Electronic, Electrical and Communication Engineering, University of Chinese Academy of Sciences; Beijing 100049, China; liuzhenxi17@mails.ucas.ac.cn (Z.L.); jmchen@mail.ie.ac.cn (J.C.); 2 State Key Laboratory of Transducer Technology, Aerospace Information Research Institute, Chinese Academy of Sciences, Beijing 100010, China

**Keywords:** piezoelectric cantilever, different width layers, tip displacement, resonance frequency

## Abstract

The piezoelectric cantilever resonator is used widely in many fields because of its perfect design, easy-to-control process, easy integration with the integrated circuit. The tip displacement and resonance frequency are two important characters of the piezoelectric cantilever resonator and many models are used to characterize them. However, these models are only suitable for the piezoelectric cantilever with the same width layers. To accurately characterize the piezoelectric cantilever resonators with different width layers, a novel model is proposed for predicting the tip displacement and resonance frequency. The results show that the model is in good agreement with the finite element method (FEM) simulation and experiment measurements, the tip displacement error is no more than 6%, the errors of the first, second, and third-order resonance frequency between theoretical values and measured results are 1.63%, 1.18%, and 0.51%, respectively. Finally, a discussion of the tip displacement of the piezoelectric cantilever resonator when the second layer is null, electrode, or silicon oxide (SiO_2_) is presented, and the utility of the model as a design tool for specifying the tip displacement and resonance frequency is demonstrated. Furthermore, this model can also be extended to characterize the piezoelectric cantilever with n-layer film or piezoelectric doubly clamped beam.

## 1. Introduction

The piezoelectric cantilever is one of the most important structures for micro-electro-mechanical system (MEMS) application and it has been used in atomic force microscopes, chemical sensors and biosensors, radio frequency (RF) switches, photon detectors, micromirrors, and energy harvesters [1,2,3,4,5,6,7]. Tip displacement and resonance frequency are two important characters of the piezoelectric cantilever, especially when setting the height of the cantilever in the RF switch [8] and tuning the resonance frequency of the energy harvester that matches the ambient vibrating frequency for improving the power efficiency [4].

The static and dynamic analyses of the piezoelectric cantilever are typically performed using the principle of strain compatibility and force-moment equilibrium employed by Timoshenko [9]. Predecessors have proposed many models to characterize the piezoelectric cantilever and guide the design [10,11,12,13,14,15,16,17,18]. Smits et al. investigated the behavior of piezoelectric bimorphs for various mechanical boundary conditions based on the thermodynamic equilibrium and derived a set of constituent equations that can be adapted to any condition. This allows us to use the bimorph as a black box, without having to consider its internal movement or charges [10]. Except for bimorphs, the constituent equations of the three-layer piezoelectric cantilever actuator with various external loads and geometry were proposed by Mieczkowski [15]. Furthermore, the multilayer model that can be used for predicting the tip displacement of the piezoelectric cantilever actuator with an arbitrary configuration of elastic and piezoelectric layers was proposed by Doeve and Huang [11,19]. Edqvist et al. established a general theoretical model to study the quasi-static and dynamic electromechanical response of the piezoelectric multilayer cantilever that was assumed to be lossless and have a linear piezoelectric response. In particular, Erturk and Inman investigated the electromechanical model based on the Euler–Bernoulli beam assumptions for predicting the response of the multilayer piezoelectric energy harvester under harmonic base excitation [20] and the frequency dependent viscoelastic dynamics of a multifunctional composite structure from finite element analysis and experimental validation [21]. In addition, they carried out a great amount of work on the static and dynamic response and equivalent electrical circuit of the nonlinear piezoelectric structure [22,23,24,25]. It is worth emphasizing that all models mentioned above assume the width of every layer is the same, then deduce the tip displacement and resonance frequency of the device.

**Figure 1:**
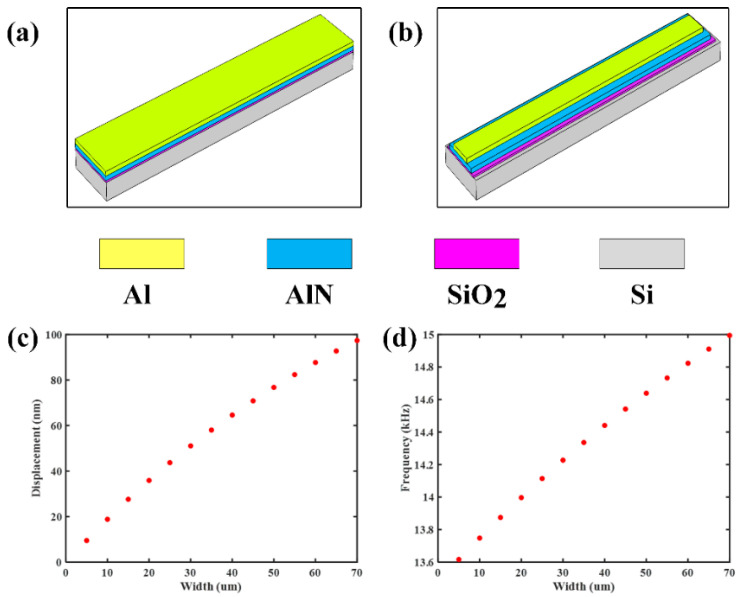
The comparison between the classical equivalent-width beam and the proposed four-layer variable width beam, and four-layer films are Al electrode, AlN piezoelectric layer, silicon oxide (SiO_2_), and the silicon substrate. (**a**) The geometry of equivalent-width beam; (**b**) the geometry of proposed variable width beam, the width gradually decreases from bottom to top; (**c**,**d**) tip displacement and fundamental frequency versus the width of the piezoelectric layer when the piezoelectric bimorph consists of Si with a width of 70 µm AlN.

However, the actual device is fabricated in different widths due to the mandatory level-to-level design rules, or the special distribution of drive and sense electrodes [26]. Figure 1a,b shows the tip displacement and fundamental frequency versus the width of the piezoelectric layer when the piezoelectric bimorph consists of Si with a width of 70 μm and aluminum nitride (AlN) with different widths, and the tip displacement and fundamental frequency increase with increasing the width of AlN. However, the tip displacement and fundamental frequency are independent of the width of the device in the classical model, which means the characteristics of these devices with different widths could not be obtained accurately by using the model with equivalent width.

The motivation of this work is to develop a model for piezoelectric multimorph cantilevers with different width layers, which can be used to characterize the tip displacement and resonance frequency. In this work, the theoretical and simulated results of six devices with different geometries are compared and the utility of the model as a design tool for specifying the tip displacement and resonance frequency is demonstrated.

## 2. Piezoelectric Cantilever Resonator Model

### 2.1. Geometry and Structure of Piezoelectric Cantilever Resonator

**Figure 2:**
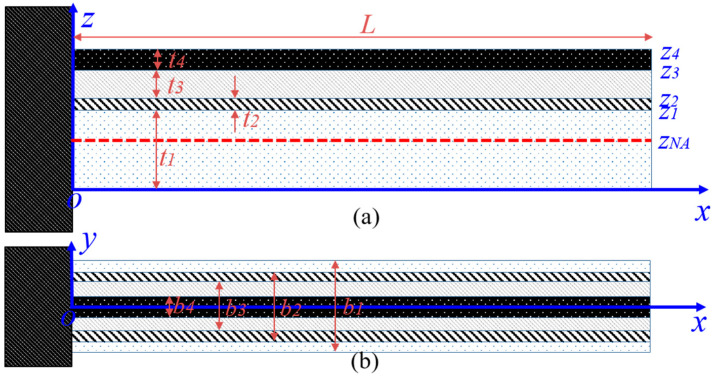
Definition of the coordinate of the four-layer piezoelectric cantilever—the first layer is the substrate, the third layer is the piezoelectric layer, and the second and fourth layer are electrodes; (**a**) side-view; (**b**) top-view.

In the actual manufacturing process of the piezoelectric cantilever, such as the standard process of MEMSCAP, the piezoelectric cantilever usually includes four-layer or three-layer film. To design and manufacture a piezoelectric cantilever that meets the requirements, a four-layer piezoelectric cantilever will be investigated in this paper. The basic geometry of a four-layer multimorph piezoelectric cantilever is shown in Figure 2. The second and fourth layers are electrode, the first layer is the substrate, and the third layer is the piezoelectric layer. The thickness and width, and length of the film are denoted by “t”, “b”, and “L”. The polarization direction of the piezoelectric layer is parallel to the *z*-axis, and the length and width direction is denoted by the *x*-axis and *y*-axis.

### 2.2. Assumptions and Theoretical Basis of the Model

The piezoelectric cantilever is subjected to a voltage, which will be deformed due to the inverse piezoelectric effect. In the formulation of this model, there are three assumptions that are critical. First, shear effects, residual stress-induced curvature, and second-order effects, such as electrostriction, are ignored; beam thickness is much less than the piezoelectric-induced curvature. Second, the width and thickness of different layers are very small compared to the length, which means that the displacement and stress along the *y*-axis and *z*-axis are equal to zero. The last assumption is that the fringe electric field of the piezoelectric layer is ignored, and there is no free charge inside the piezoelectric layer. Based on the assumptions above, the constitutive piezoelectric equations reduce as below [10].

(1) $$S_{x}=s_{1}T_{x}\;+\;d_{31}E_{z},\;D_{z}=d_{31}T_{x}+\varepsilon_{3}E_{z}$$

where $S_{x}$ and $T_{x}$ are the strain and stress in the *x*-direction of the beam, $D_{z}$ and $E_{z}$ are the electrical displacement and electric field across the piezoelectric film in the z-direction, and $s_{1}$, $d_{31}$, and $\varepsilon_{3}$ are the *x*-axis compliance, transverse piezoelectric coefficient, and permittivity of the piezoelectric film, respectively. Additionally, the electric displacement divergence is equal to zero [17], that is

(2) ∇·D=0

For the *i*-th layer film, Hooke’s law gives

(3) $$T_{i}=Y_{i}S_{i}$$

where $T_{i}$, $Y_{i}$, and $S_{i}$ are the stress, Young’s modulus, and strain of the *i*-th layer film.

The strain can be written as

(4) $$S_{x}=-z\frac{\partial^{2}w}{\partial x^{2}}$$

where *w* is the displacement of the piezoelectric cantilever. As all films are closely linked, their displacement and the strain are the same.

### 2.3. Tip Displacement

The static displacement of the piezoelectric cantilever was deduced using the general Bresse–Kirchhoff–Timoshenko beam theory, which is based on the assumptions of straightness and inextensibility. It takes into account the shear deformation and rotational bending effects, making it suitable for describing the behavior of composite beams. In fact, all derivations are based on the beam’s force and moment balance. Before using the moment equilibrium, the neutral plane where the stress is equal to zero will be calculated. The determination of the neutral plane position is carried out by integrating the stress across the surface of the beam section:

(5) $$N=\overset{4}{\underset{i=1}{\sum }}\iint^{\;}T_{i}dA=0$$

Combining with (3), then:

(6) $$N=\overset{4}{\underset{i=1}{\sum }}\int_{z_{i-1}}^{z_{i}}Y_{i}\left(z-z_{NA}\right)b_{i}dz=0$$

In addition, the neutral plane position can be obtained as below:

(7) $$z_{NA}=\frac{\sum_{i=1}^{4}Y_{i}b_{i}t_{i}\left(z_{i}\;+\;z_{i-1}\right)}{2\sum_{i=1}^{4}Y_{i}b_{i}t_{i}}=\frac{\sum_{i=1}^{4}Y_{i}b_{i}t_{i}\left(\frac{t_{i}}{2}+\sum_{j=1}^{i-1}t_{j}\right)}{\sum_{i=1}^{4}Y_{i}b_{i}t_{i}}$$

where $b_{i}$ and $t_{i}$ are the width and thickness of the *i*-th layer film, $z_{i}$ is the ordinary of the upper surface of the *i*-th layer film, which equals the total thickness of the front *i* layer, and we note $z_{0}=0$.

Similarly, the sum of moments can be obtained by integrating the stress multiplied by the distance to the neutral plane across the surface of the beam section:

(8) $$M=\overset{4}{\underset{i=1}{\sum }}\iint^{\;}T_{i}zdA$$

Combining with (1) and (3), then

(9) $$M=\overset{}{\underset{i=1,2,4}{\sum }}\int_{z_{i-1}-z_{NA}}^{z_{i}-z_{NA}}Y_{i}S_{i}b_{i}zdz\;+\;\int_{z_{2}-z_{NA}}^{z_{3}-z_{NA}}\left(Y_{3}S_{3}b_{3}-d_{31}Y_{3}E_{3}b_{4}\right)zdz$$

(10) $$E_{3}=\frac{V}{t_{3}}$$

where $E_{3}$ and *V* are the electric field and voltage across the piezoelectric film. It is noticed that the effective area of the piezoelectric layer with applied voltage is determined by the top electrode, not its area. That is, the piezoelectric moment is relative to the width of the top electrode. It is different from the piezoelectric cantilever with the same width layers.

Rearrange (9) and we can obtain

(11) $$M=\overset{4}{\underset{i=1}{\sum }}\int_{z_{i-1}-z_{NA}}^{z_{i}-z_{NA}}Y_{i}S_{i}b_{i}zdz-\int_{z_{2}-z_{NA}}^{z_{3}-z_{NA}}d_{31}Y_{3}E_{3}b_{4}zdz=\overset{4}{\underset{i=1}{\sum }}M_{i}-M_{p}$$

(12) $$M_{i}=\int_{z_{i-1}-z_{NA}}^{z_{i}-z_{NA}}Y_{i}S_{i}b_{i}zdz=-\frac{\partial^{2}w}{\partial x^{2}}Y_{i}b_{i}\int_{z_{i-1}-z_{NA}}^{z_{i}-z_{NA}}z^{2}dz=-\frac{\partial^{2}w}{\partial x^{2}}K_{i}$$

(13) $$K_{i}=\frac{Y_{i}b_{i}}{3}\left[{\left(z_{i}-z_{NA}\right)}^{3}\;-\;{\left(z_{i-1}\;-\;z_{NA}\right)}^{3}\right]$$

(14) $$M_{p}=d_{31}Y_{3}E_{3}b_{4}\int_{z_{2}-z_{NA}}^{z_{3}-z_{NA}}zdz=\frac{d_{31}Y_{3}E_{3}b_{4}}{2}\left[{\left(z_{3}\;-\;z_{NA}\right)}^{2}\;-\;{\left(z_{2}\;-\;z_{NA}\right)}^{2}\right]$$

where $K_{i}$ is the flexural rigidity of the *i*-th layer film, $M_{i}\;$is the moment generated by the i-th layer film, and $M_{p}$ is the piezoelectric moment, which is the origin of the cantilever bending. Utilizing the moment equilibrium, that is, the total moment is equal to zero [16].

(15) $$M=-\frac{\partial^{2}w}{\partial x^{2}}K-M_{p}=0$$

(16) $$K=\sum_{i=1}^{4}K_{i}$$

Solving the equation above, the static displacement can be calculated as below

(17) $$w\left(x\right)=-\frac{M_{p}}{2K}x^{2}$$

The tip displacement can be obtained by letting x=L, hence

(18) $$d=-\frac{M_{p}}{2K}L^{2}$$

where *L* is the length of the piezoelectric cantilever, and the negative sign indicates that the piezoelectric cantilever is bent downward. From (13), (14), and (18), the tip displacement is relative to the width of every film. In addition, the tip displacement and the voltage across the piezoelectric film have a linear relationship.

**Figure 3:**
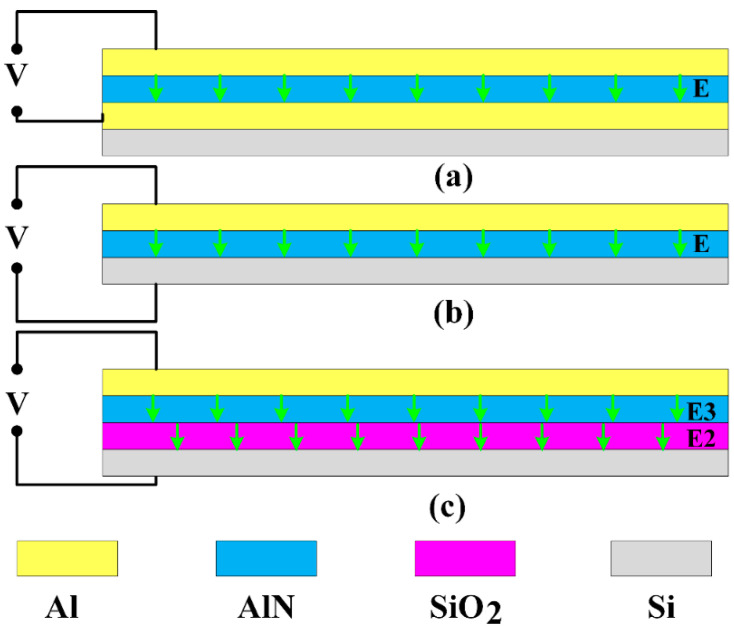
The simplified schematics of configurations of the applied voltage when the second layer is electrode, null, and SiO_2_. (**a**) The second layer is electrode, the top and bottom electrode are connected with the voltage and ground; (**b**) the second layer is null, that is there are only three layers: top electrode, piezoelectric layer, and substrate. The top electrode and the substrate are connected with voltage and ground; (**c**) the second layer is SiO_2_, the top electrode and the substrate are connected with voltage and ground.

For most piezoelectric devices, the second layer is the electrode. However, there are some situations that the second layer is null (only three layers) or the second layer is silicon oxide (SiO_2_), as shown in Figure 3.

In most cases, the piezoelectric cantilever consists of top electrode, piezoelectric layer, bottom electrode, and the substrate. The top electrode and bottom electrode are connected with voltage and ground, respectively, making the cantilever bent, which has been analyzed and discussed above. In some applications where large displacement is desired, the bottom electrode does not need to be designed and fabricated, so that the second layer is null. In this case, the voltage and ground are applied between the top electrode and the substrate. The electric field across the piezoelectric layer is the same as that of the aforementioned case, which equals to V/t3, where t3 is the thickness of piezoelectric layer. In addition, the second layer could be SiO_2_ to prevent leakage and breakdown, as shown in Figure 3c; though the electrical connection is the same as the second situation, the actual voltage applied on the piezoelectric layer will be decreased due to the existence of SiO_2_, and the actual electric field across the piezoelectric layer is determined by the permittivity of AlN and SiO_2_. Therefore, the analysis of the first case can be used to analyze the second case similarly; just use a three-layer film model instead. However, for the third case, using the continuity of the electrical displacement of different dielectrics, the electric displacement of AlN is equal to that of SiO_2_. After the electric field applied on the piezoelectric layer is obtained, the analysis of the first case can also be appropriate for this case.

To find the actual electric field across the piezoelectric layer, the continuity of electrical displacement of different dielectrics is utilized. According to the continuity of electrical displacement, the electric displacement of AlN is equal to that of SiO_2_, that is,

(19) $$D_{2}=D_{3}$$

(20) $$E_{2}\varepsilon_{2}=\varepsilon_{3}E_{3}$$

where $D_{2}\;$and $D_{3}$ are the electric displacement of SiO_2_ and AlN, $E_{2}\;$and $E_{3}$ are the electric field across SiO_2_ and AlN, $\varepsilon_{2}\;$and $\varepsilon_{3}$ are the permittivity of SiO_2_ and AlN. On the other hand, the applied voltage is equal to the sum of the voltage drop in AlN and the voltage drop in SiO_2_, that is,

(21) $$E_{2}t_{2}\;+\;E_{3}t_{3}=V$$

Combing (20) and (21), we can obtain the electric field across AlN as below

(22) $$E_{3}=\frac{V}{t_{3}\;+\;\varepsilon_{3}t_{2}/\varepsilon_{2}}$$

After obtaining the electric field across the piezoelectric film, the tip displacement can be calculated using (18).

### 2.4. Resonance Frequency

The motion equation of bending piezoelectric cantilever is given by Newton’s law:

(23) $$m\frac{\partial^{2}w}{\partial t^{2}}=\frac{\partial^{2}M}{\partial x^{2}}$$

(24) $$m=\overset{4}{\underset{i=1}{\sum }}\rho_{i}b_{i}t_{i}$$

where m is the mass of the unit length of the piezoelectric cantilever, and $\rho_{i}$ is the mass density of the *i*-th layer film. Substituting (11) into (23), then

(25) $$K\frac{\partial^{4}w}{\partial x^{4}}\;+\;m\frac{\partial^{2}w}{\partial t^{2}}=0$$

Applying the separated variable method and combining the boundary conditions of cantilever [14], we can obtain the resonance frequency and modal function as below:

(26) $$f_{i}=\frac{\beta_{i}^{2}}{2\pi L^{2}}\sqrt{\frac{K}{m}}$$

(27) $$\phi_{i}\left(x\right)=\sigma_{i}\left[sin\left(\beta_{i}x\right)-sinh\left(\beta_{i}x\right)\;+\;\gamma_{i}\left(cos\left(\beta_{i}x\right)\;-\;\cos h(\beta_{i}x\right))\right]$$

where $\beta_{i}$, $\sigma_{i}$, and $\gamma_{i}$ are coefficients of modal function. For the cantilever, these three coefficients of the first, second, and third-order modal function are listed in Table 1 [17]. For example, the foundational frequency is given by

(28) $$f_{1}=\frac{1.8746^{2}}{2\pi L^{2}}\sqrt{\frac{K}{m}}$$

## 3. Device and Fabrication

**Figure 4:**
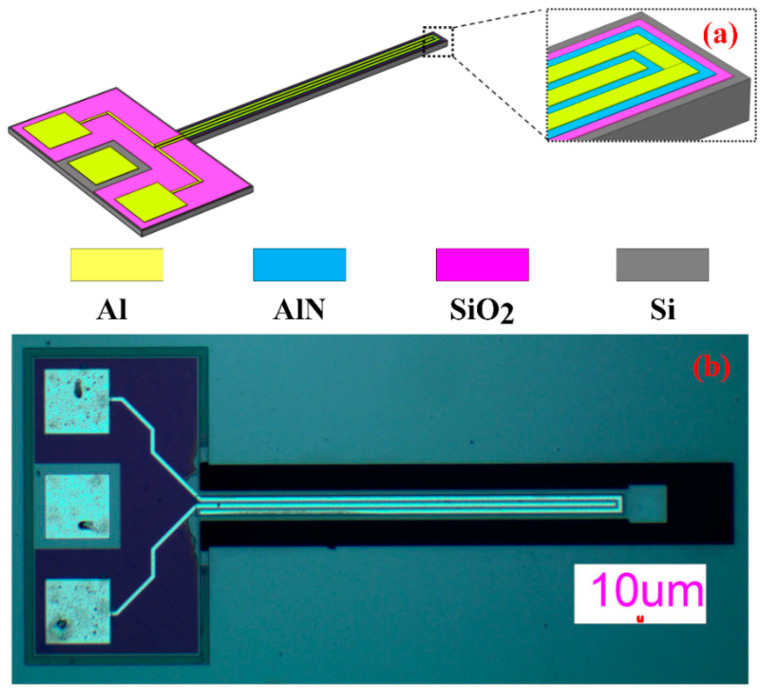
The layout of the piezoelectric cantilever. (**a**) Three-dimensional structure; (**b**) the microscope image of one of the piezoelectric cantilevers fabricated using the MEMSCAP piezo micromachining process.

The piezoelectric cantilever structures were fabricated using the standard process of MEMSCAP to compare the theoretical model and experimental results. Figure 4 shows the layout of the piezoelectric cantilever. The total fabrication was a five-masks process. In this fabrication process, the substrate is 400 μm silicon layer and a silicon-on-insulator (SOI) (10 μm silicon and 1 μm oxide), the second layer is a thin thermal oxide layer (0.2 μm), which means there is no bottom electrode layer, so the silicon in SOI grounded as the bottom electrode. Then a 0.5 μm thin aluminum nitride (AlN) was deposited and patterned as the piezoelectric layer, and a metal stack consisting of 20 nm chrome and 1 μm aluminum (Al) was deposited and patterned using the lift-off process to define the top electrode layer, connecting wire, and contact electrode. Finally, the piezoelectric cantilever was released by two-step deep reaction ion etching (DRIE) and wet oxide etch process [26].

Figure 4b is the high magnification microscope image of one of the fabricated devices by PiezoMUMPs process, the length of the piezoelectric cantilever is 1100 μm, and the widths of Si, SiO_2_, AlN, and Al are 70 μm, 60 μm, 50 μm, and 30 μm. The geometry parameters are listed in Table 2.

## 4. Results and Discussion

### 4.1. Measurements and Results

**Figure 5:**
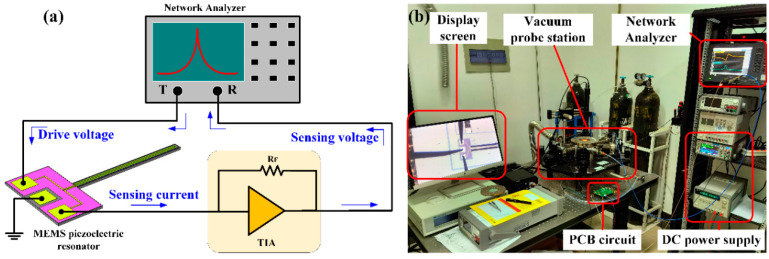
The schematic of the open-loop electrical measurement setup. (**a**) The drive voltage generated by a Network Analyzer is applied in the driving electrode (left); the middle electrode connects the ground; then, the sensing current generated in the sensing electrode (right) was fed to the transimpedance amplifier (TIA), which consists of an operational amplifier and a feedback resistance, transforming the current into voltage; finally, the sensing voltage is fed to the Network Analyzer. The Network Analyzer will automatically display the transmission curve, as shown in Figure 6. The blue arrows represent the signal flow; (**b**) the actual measurement setup, whereby the piezoelectric cantilever is inside the vacuum probe station, which is amplified by the microscope and is shown in the display screen. The black probes in the display screen are sensing, driving, and ground electrode.

The interface circuit for measuring the resonance frequency of the piezoelectric cantilever resonator is described in Figure 5, which shows a trans-impedance amplifier (TIA) (also called charge amplifier) to change the current (charge) to voltage. The measurement of electrical transmission of piezoelectric cantilever resonator was carried out by using the network analyzer (E5061B). The input signal was fed to the input electrode, and the sensed signal was fed to the network analyzer from the sensing electrode, and the middle electrode was grounded [27].

**Figure 6:**
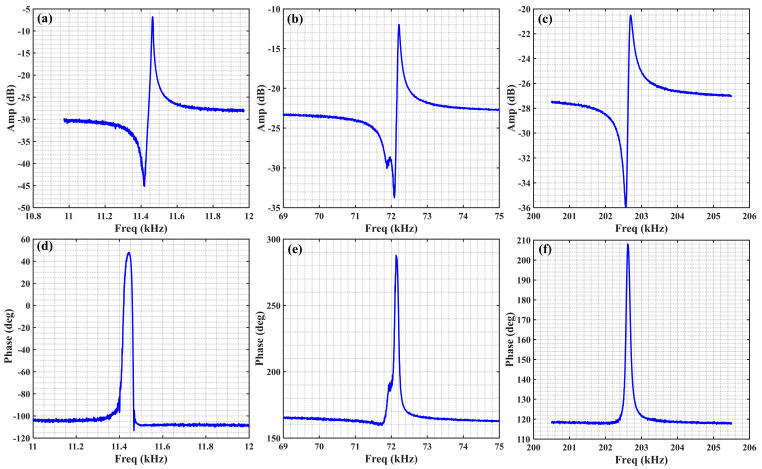
Measurements of S21 of the fabricated piezoelectric cantilever resonator, the 1st, 2nd, and 3rd-order resonance frequency are 11.463 kHz, 72.208 kHz, and 202.701 kHz, respectively. (**a–c**) The frequency–amplitude response curve of the 1st, 2nd, and 3rd-order resonance mode; (**d–f**) the frequency–phase response curve of the 1st, 2nd, and 3rd-order resonance mode.

**Figure 7:**
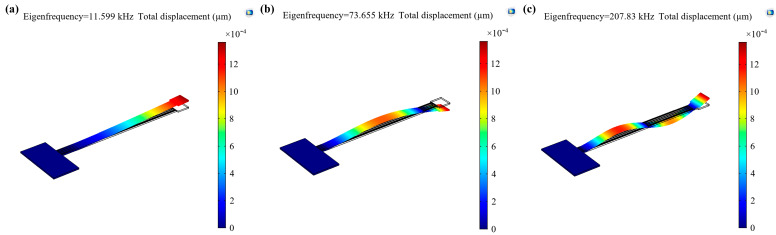
The eigenfrequency of the 1st, 2nd, and 3rd-order resonance mode with COMSOL Multiphysics simulation. (**a**) The first-order resonance frequency, 11.599 kHz; (**b**) the second-order resonance frequency, 73.655 kHz; (**c**) the third-order resonance frequency, 207.830 kHz.

The transmission characteristics of the piezoelectric cantilever resonator are shown in Figure 6. The first, second, and third-order resonance frequency are 11.463 kHz, 72.208 kHz, and 202.701 kHz. According to the proposed model, using the geometry and material parameters of the piezoelectric cantilever resonator listed in Table 2, the first, second, and third-order resonance frequency are 11.654 kHz, 73.071 kHz, and 201.66 kHz; the errors between theoretical values and measured results are 1.63%, 1.18%, and 0.52%. Additionally, the resonance mode and eigenfrequency with finite element method (FEM) simulation are shown in Figure 7; the first, second, and third-order resonance frequency are 11.599 kHz, 73.655 kHz, and 207.83 kHz; the errors between simulated values and measured results are 1.17%, 1.96%, 2.47%, respectively; the errors between simulated values and theoretical values are 0.47%, 0.80%, and 3.06%; the comparison of them is listed in Table 3. All errors are quite small, so we can conclude that the utility of the model is demonstrated; meanwhile the FEM simulation is also a useful method to obtain the real resonance frequency of piezoelectric cantilever resonator.

**Figure 8:**
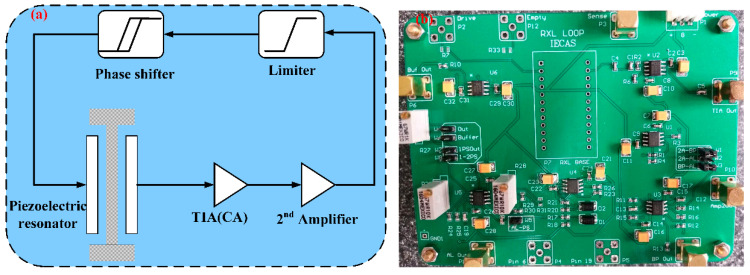
The closed-loop interface circuit of the piezoelectric cantilever resonator for measuring the tip displacement. (**a**) The schematic of interface circuit; (**b**) the actual PCB circuit.

To measure the tip displacement of the piezoelectric cantilever resonator, the closed-loop interface circuit is designed, as shown in Figure 8. It consists of a piezoelectric cantilever resonator, TIA, the second operational amplifier, amplitude limiter, and phase shifter. The TIA converts the motion current signal of the resonator into a voltage signal; the second operational amplifier further amplifies the voltage signal output by the TIA so that the loop meets the amplitude condition of the Barkhausen criterion. The amplitude limiter is a controllable nonlinear device in the loop to stabilize the amplitude of the self-excited signal; the phase shifter shifts the phase of the output voltage signal of the amplitude limiter so that the loop meets the phase condition of the Barkhausen criterion [28,29,30].

The measurement principle of tip displacement is as below: Firstly, we build the closed-loop circuit, make it oscillate, and then record the drive voltage (Vd) and the output voltage (V2) of the second operational amplifier. Second, combining the output voltage of the second operational amplifier, feedback resistance (Rf) in the TIA, and the gain (G) of the second operational amplifier, we calculate the motion current (I). Finally, we find the electromechanical coupling coefficient (η) (see Appendix A) [31], and utilie both it and the resonance frequency to obtain the tip displacement when the piezoelectric cantilever is at resonance, which is *Q* (quality factor) times the static tip displacement.

(29) $$I=\frac{V_{2}}{G\cdot R_{f}}$$

(30) $$x_{r}=\frac{I}{2\pi f_{r}\eta }$$

(31) $$d=\frac{x_{r}}{Q}$$

**Figure 9:**
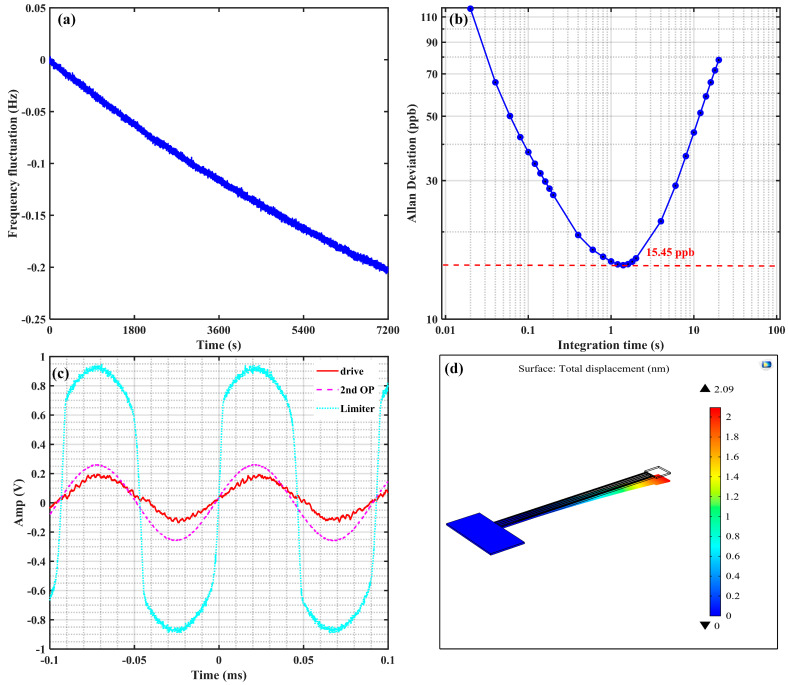
The characteristics of close-loop oscillation of piezoelectric cantilever resonator and the simulated result. (**a**) The frequency fluctuation of the resonator within two hours—the resonance frequency is reduced by 0.2 Hz; (**b**) the Allan deviation of the resonator within two hours—the minimal Allan deviation is 15.45 ppb; (**c**) the time domain wave of the output voltage of the driving port, the 2nd operational amplifier, and amplitude limiter; (**d**) the tip displacement with FEM simulation—the tip displacement is 2.09 nm.

When the piezoelectric cantilever resonator oscillates stably, we use the frequency counter to sample the frequency value continuously for two hours and obtain its Allan deviation, as shown in Figure 9a, the minimal Allan deviation is 15.45 ppb, which indicates that the resonator can oscillate very stably. The drive signal and the output voltage of the second operational amplifier are 0.2 V and 0.25 V, as shown in Figure 9b. In the PCB circuit, the feedback resistance and gain of the second operational amplifier are 300 kΩ and 100, respectively. According to (29), the motion current is 8.33 nA. From the analysis in Appendix A, the electromechanical coupling coefficient η is $5.071\times 10^{-8}$, and the resonance frequency is 11.463 kHz, then the tip displacement is 2.282 μm. In the open-loop test, the quality factor of the piezoelectric cantilever resonator is 1411.2, therefore, the static tip displacement is 1.62 nm.

Back to the proposed model, let the applied voltage be the drive voltage, that is 0.2 V. Substituting the geometry and material parameters to (1–18), the static tip displacement is 1.88 nm, which is larger than the measured result. The reason is that there is a feedthrough capacitor in the resonator [32], which will affect the output current. On the other hand, the nonideal factors exist in the PCB circuit, such as parasitic parameters of electrical elements. Additionally, the simulated value with COMSOL Multiphysics is 2.09 nm, which is close to the theoretical value. Comparing these three results, although there are some differences, they are relatively close and can be used to predict the tip displacement of the piezoelectric cantilever resonator.

### 4.2. Discussion

To compare the proposed model with the models proposed by predecessors, the tip displacement of six piezoelectric cantilevers with different widths were simulated with COMSOL Multiphysics. The geometry and material parameters are listed in Table 4. The piezoelectric cantilever is actuated with the DC voltage 1 V. The tip displacement of the newly proposed model and that obtained utilizing the model in the literature [11,17] were compared, and the error between the simulated results and theoretical values is calculated, as shown in Table 5. It is shown that the error of the newly proposed model was the smallest and its error is no more than 6%. It is noted that if the width of the piezoelectric layer and the top electrode is the same, the displacement calculated with [11] and with the newly proposed model will also be the same; the reason for this is that the width of the piezoelectric layer subjected to voltage is the width of the top electrode.

In some applications, there are some requirements for the tip displacement of the piezoelectric cantilever. Therefore, the paper will discuss the tip displacement of the piezoelectric cantilever driven by the same voltage when the second layer is null, electrode, and SiO_2_. Assuming that the devices have the same geometry, the simulated tip displacement and the theoretical values are listed in Table 6 and Table 7. The results show the error is no more than 7%, which means that the theoretical analysis can be used to describe the tip displacement of the piezoelectric cantilever. Additionally, compared to three situations about the second layer, the displacement is the largest when the second layer is null, and the displacement is the smallest when the second layer is SiO_2_ and the substrate grounded. Therefore, if we want a larger tip displacement for a given size, removing the second layer will be the optimal structure.

## 5. Conclusions

A model used to characterize the tip displacement and resonance frequency of piezoelectric cantilever with different width layers was proposed in this paper. The experiment measurements and FEM simulation results agree well with the model, the tip displacement error is no more than 6 %, the tip displacement error deduced by the newly proposed model is smaller than predecessors’ models. The errors of the first, second, and third-order resonance frequency between theoretical values and measured results are 1.63%, 1.18%, and 0.52%, respectively.

In addition, the model proposed can accurately deduce the tip displacement when the piezoelectric cantilever is fabricated in three situations: the second layer is SiO_2_, electrode, or null. The maximum error of the tip displacement among these three situations is no more than 7%. When the devices fabricated in these three situations have the same geometry, and are applied in the same voltage, the tip displacement is the largest when the second layer is null, and the tip displacement is the smallest when the second layer is SiO_2_. Therefore, if one wants a larger tip displacement for a given size, removing the second layer will be the optimal structure.

In the paper, we have verified that the model can be adapted to the three-layer beam and four-layer beam. Thus, we can extend this model to a n-layer piezoelectric cantilever with different widths, but every piezoelectric layer must have two metal layers for applying the voltage. Additionally, this model can also be used to characterize the piezoelectric doubly fixed beam with the same length—especially the drive electrode must have the same length with the piezoelectric layer, otherwise the model will not meet the linear assumption of the model. The extensive work will be studied in our future work.

## Figures and Tables

**Table 1:** Three coefficients of the modal function.

Mode	$\sigma_{i}$	$\beta_{i}$	$\gamma_{i}$
1st mode	0.3669	1.8746	−1.3629
2nd mode	−0.5093	4.6927	−0.9818
3rd mode	0.4996	7.8500	−1.0080

**Table 2:** Geometry and material parameters of the piezoelectric resonator.

Layer	t (µm)	b (µm)	L (µm)	Y (GPa)	υ	*ρ* (kg/m^3^)	*ε*
1: Si	10	70	1100	160	0.22	2320	4.5
2: SiO_2_	0.2	60	1100	70	0.17	2700	4.2
3: AlN	0.5	50	1100	345	0.32	3300	9
4: Al	1.0	30	1100	70	0.33	2700	-
	Piezoelectric coupling coefficient: d31 = −1.9159 × 10^−12^ C/N						

**Table 3:** Comparison of theory, simulation and measurement of resonance frequency.

Freq.	Simulation (KHz) (1)	Measured (KHz) (2)	Proposed Model (3)	Error (%) (1) and (2)	Error (%) (2) and (3)	Error (%) (1) and (3)
1st order	11.599	11.463	11.654	1.17	1.63	0.47
2nd order	73.655	72.208	73.071	1.96	1.18	0.80
3rd order	207.83	202.701	201.66	2.47	0.52	3.06

**Table 4:** Geometry and material parameters of the piezoelectric resonator.

Layer	t (µm)	L (µm)	Y (GPa)	υ	*ρ* (kg/m^3^)	ε
1: Si	10	1000	160	0.22	2320	4.5
2: Al	0.2	1000	70	0.33	2700	-
3: AlN	0.5	1000	345	0.32	3300	9
4: Al	1.0	1000	70	0.33	2700	-
Piezoelectric coupling coefficient: d31 = −1.9159 × 10^−12^ C/N						

**Table 5:** Comparison of tip displacement of predecessor’s model and newly proposed model.

Test	b1 (µm)	b2 (µm)	b3 (µm)	b4 (µm)	Ref [11] (nm)	Ref [17] (nm)	Proposed (nm)	FEM (nm)	Error (%)
#1	70	70	70	70	88.42	78.83	88.42	88.77	0.39
#2	70	70	70	20	93.04	23.70	26.58	27.70	4.04
#3	70	70	20	20	35.08	31.28	35.08	34.35	2.13
#4	70	20	20	20	35.82	31.94	35.82	34.98	2.40
#5	70	50	30	10	50.50	15.01	16.83	17.75	5.18
#6	70	60	50	10	74.41	13.32	14.94	15.89	5.98

**Table 6:** Tip displacement when the second layer is null.

Test	b1 (µm)	b3 (µm)	b4 (µm)	Proposed (nm)	FEM (nm)	Error (%)
#1	70	70	70	88.95	89.70	0.84
#6	70	50	10	14.95	15.94	6.21

**Table 7:** Tip displacement when the second layer is SiO_2_ and the substrate grounded.

Test	b1 (µm)	b2 (µm)	b3 (µm)	b4 (µm)	Proposed (nm)	FEM (nm)	Error (%)
#1	70	70	70	70	47.61	46.07	3.34
#6	70	60	50	10	8.04	8.54	5.85

## Data Availability

Data is contained within the article. More detailed data and data presented in this study are available on request from the corresponding author. Part of them could be included in the Final reports to the corresponding funding organizations.

## Appendix A

For a piezoelectric cantilever resonator, the electromechanical coupling coefficient (*η*) at the sensing port can be defined as

(A1) $$\eta =\frac{Q\left(j\omega \right)}{W\left(j\omega \right)}=\frac{I\left(j\omega \right)}{j\omega W\left(j\omega \right)}$$

where *Q* and *I* are the output charge and current, *W* is the generalized cantilever displacement, ω is the resonance angular frequency, η has the unit of (C/m). The charge can be deduced by integrating the piezoelectrically induced charge per unit, $D_{3}$, over the area of the sensing electrode:

(A2) $$I=j\omega Q=j\omega \int_{0}^{L}\int_{0}^{b4}D_{3}dxdy=j\omega b_{4}\int_{0}^{L}e_{31f}T_{3}dx=j\omega b_{4}e_{31f}\int_{0}^{L}z_{p}\frac{\partial^{2}w}{\partial x^{2}}dx$$

(A3) $$z_{p}=\frac{{\left(z_{3}\;-\;z_{NA}\right)}^{2}\;-\;{\left(z_{2}\;-\;z_{NA}\right)}^{2}}{2}$$

where $e_{31f}$ is the piezoelectric coupling coefficient, e31f=−1.08 Cm2. According to the analysis of Section 2.4, the displacement of the piezoelectric cantilever can be described as the product of tip displacement and the modal function.

(A4) w=W·ϕ(x)

Substituting (A4) to (A2), then

(A5) $$I=\;\frac{j\omega b_{4}e_{31f}z_{p}W}{L}\int_{0}^{1}\frac{\partial^{2}\phi }{\partial x^{2}}dx$$

Therefore, the electromechanical coupling coefficient (η) can be obtained as below:

(A6) $$\eta =\frac{I}{j\omega W}=\frac{b_{4}e_{31f}z_{p}}{L}\int_{0}^{1}\frac{\partial^{2}\phi }{\partial x^{2}}dx$$
